# Synthetic Nanoparticles That Promote Tumor Necrosis Factor Receptor 2 Expressing Regulatory T Cells in the Lung and Resistance to Allergic Airways Inflammation

**DOI:** 10.3389/fimmu.2017.01812

**Published:** 2017-12-22

**Authors:** Rohimah Mohamud, Jeanne S. LeMasurier, Jennifer C. Boer, Je Lin Sieow, Jennifer M. Rolland, Robyn E. O’Hehir, Charles L. Hardy, Magdalena Plebanski

**Affiliations:** ^1^Department of Immunology and Pathology, Monash University, Melbourne, VIC, Australia; ^2^CRC for Asthma and Airways, Sydney, NSW, Australia; ^3^Department of Immunology, School of Medical Sciences, Universiti Sains Malaysia, Kelantan, Malaysia; ^4^Department of Allergy, Immunology and Respiratory Medicine, Monash University and The Alfred Hospital, Melbourne, VIC, Australia; ^5^School of Health and Biomedical Sciences, RMIT, Melbourne, VIC, Australia

**Keywords:** nanoparticles, tumor necrosis factor 2, asthma, PS50G, lung, lymph nodes, animal model

## Abstract

Synthetic glycine coated 50 nm polystyrene nanoparticles (NP) (PS50G), unlike ambient NP, do not promote pulmonary inflammation, but instead, render lungs resistant to the development of allergic airway inflammation. In this study, we show that PS50G modulate the frequency and phenotype of regulatory T cells (Treg) in the lung, specifically increasing the proportion of tumor necrosis factor 2 (TNFR2) expressing Treg. Mice pre-exposed to PS50G, which were sensitized and then challenged with an allergen a month later, preferentially expanded TNFR2^+^Foxp3^+^ Treg, which further expressed enhanced levels of latency associated peptide and cytotoxic T-lymphocyte associated molecule-4. Moreover, PS50G-induced CD103^+^ dendritic cell activation in the lung was associated with the proliferative expansion of TNFR2^+^Foxp3^+^ Treg. These findings provide the first evidence that engineered NP can promote the selective expansion of maximally suppressing TNFR2^+^Foxp3^+^ Treg and further suggest a novel mechanism by which NP may promote healthy lung homeostasis.

## Introduction

Nanoparticles (NP), defined as particles with a diameter less than 100 nm, comprise the dominant type of particles in ambient airborne particulate matter (1). NP can be divided into three categories: naturally occurring, anthropogenic, and engineered nanoparticles (ENP) that are manufactured for industrial or consumer applications (2). The increasing use of NP for pulmonary drug delivery (3, 4) continues to drive the debate on their potential to negatively or positively modulate lung immune homeostasis (5).

The lung is confronted by a diverse range of natural and man-made NP, on a daily basis, and must maintain a state of immune ignorance or “tolerance” to retain pulmonary homeostasis and prevent undesirable immunopathology. As nanotechnology develops, it is clear that it will also be important to understand the impact of ENP on the lung. ENP by themselves have the capability to induce beneficial or detrimental effects on lung immune homeostasis, depending on their characteristics (2). For example, nickel NP (6) and titanium dioxide NP (7) can exacerbate existing allergic airway inflammation (AAI). However, inert glycine-coated polystyrene 50-nm NP (PS50G) behave differently from most of the ubiquitous particles in the environment, *in vivo* and *in vitro* (8–12). Of note, PS50G also induce the secretion of chemokines involved in recruitment and/or maturation of monocytes and dendritic cells (DCs), and pre-exposure to PS50G prevents the subsequent elicitation of AAI (8). Furthermore, immune imprinting by PS50G in the lung leads to subsequently modified pulmonary immune responses to allergens (9).

Immune imprinting or “innate training” is a phenomenon wherefore non antigenic stimuli (e.g., toll-like receptor ligands or NP) alter the capacity of the immune system to react to subsequent unrelated stimuli (13, 14). Some innate training mechanisms include impairment of pulmonary antigen-presenting cell (APC) function (9, 15), altered antigen delivery (16), and induction of regulatory myeloid-derived suppressor cells (12). Previously, we demonstrated that PS50G not only negatively imprint inflammatory CD11b^hi^ dendritic cell (DC) but also increase the frequency of CD103^+^ DC in the lung (9), a population that contributes to airway homeostasis by inducing Foxp3^+^ regulatory T cells (Treg) (17), through a Treg-independent production of IL-10 (18) or IL-12 (19). By using AAI murine models, Treg were demonstrated to play a major role in controlling lung homeostasis and its responsiveness to environmental allergens (20, 21). Therefore, we hypothesized that PS50G innate training would also substantially change the homeostasis of Treg in the lung, particularly inflammation related Treg expressing tumor necrosis factor (TNF) receptor 2 (TNFR2^+^Foxp3^+^ Treg), reported as maximally suppressive in other disease settings (22–24).

## Materials and Methods

### Mice

Female BALB/c mice aged 6–8 weeks were obtained from the Walter and Eliza Hall Institute of Medical Research, Melbourne, VIC, Australia and housed in the Alfred Medical Research and Education Precinct (AMREP) animal house. All studies with mice were approved by the AMREP Animal Ethics Committee.

### Particle Preparation, Instillation, and Immunization

Polybead carboxylate microspheres (unlabeled, nominally 0.05 mm; no. 15913; Polysciences, Warrington, PA, USA) were glycine coated, as described (25) and referred to as PS50G. To investigate the long-term effects of PS50G on the innate immune response, mice received saline or PS50G (200 µg/50 µl) intratracheally on day 0 and lymph nodes (LN) and lungs were collected on days 1, 3, 7, and 30 post instillation. In some experiments, 10 µg lipopolysaccharide derived from *Escherichia coli* (Sigma-Aldrich, St. Louis, MO, USA) were used as a positive “inflammatory” control. The effects of PS50G on acute allergic asthma were investigated by intratracheally instilled PS50G (200 µg/50 µl) into mice on days 0 and 2 prior to intraperitoneal sensitization with ovalbumin (OVA) (50 µg; Sigma-Aldrich, St. Louis, MO, USA) in aluminum hydroxide (General Chemical, Parsippany, NJ, USA) on days 12 and 22 and intranasal OVA challenge (25 µg) on days 32, 34, 37, and 39. Tissue sampling was performed 24 h after the final lung allergen challenge (day 40) as described (8, 9).

### Antibodies, Surface, and Intracellular Staining

Cells (1 × 10^6^) were stained on ice for 20 min with combinations of the following antibodies: CD3 (APC-Cy7 and Qdot 605) (Life technologies, Grand Island, NY, USA); CD4 (V450 and V500) (BD Biosciences, San Jose, CA, USA); CD25 (PE-Cy7 and APC-Cy7), CD120b/tumor necrosis factor 2 (TNFR2) (PE), latency associated peptide (LAP) (Per-CP), cytotoxic T-lymphocyte associated molecule-4 (CTLA-4) biotin or their respective immunoglobulin isotypes (all eBioscience, San Diego, CA, USA). For intracellular staining of Foxp3 (APC) and Ki67 (FITC) (eBioscience, San Diego, CA, USA), cells were first permeabilized according to the manufacturer’s instructions. The following antibodies were used to identify CD103^+^ DC: CD103 (PE) (BD Biosciences), CD11c (APC) and MHCII (APC-eFluor 780) (eBioscience), CD11b (AF700) and CD86 (Brilliant Violet Blue) (BioLegend), and Live/Dead cell stain kit-Aqua (Invitrogen). Acquisition was on an LSR II flow cytometer (BD Biosciences, San Jose, CA, USA) and analysis was performed using FlowJo (Tree Star, Ashland, OR, USA).

### Statistical Analysis

Data were analyzed for normality and log-transformed as necessary prior to analysis by Student’s *t-*test or ANOVA with Bonferroni posttests, depending on the number of experimental groups. Spearman’s correlations were used for the comparison of continuous variables. The Spearman’s *r* value for the correlation between the two variables was stated in each result. Statistical analysis was performed using Graph Pad Prism v5.02 software. Group sizes are indicated in the figure legends. Data are expressed as mean ± SEM. *p < 0.05, **p < 0.01, and ***p < 0.001.

## Results

### PS50G Instillation Increased TNFR2^+^Foxp3^+^ Treg in the Lung

**Figure 1:**
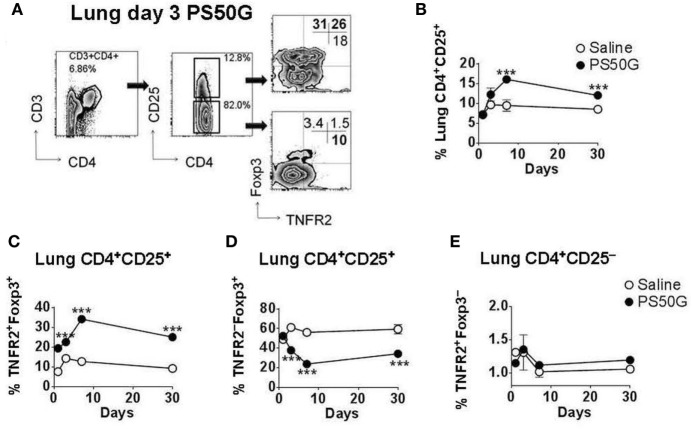
PS50G instillation selectively increases lung CD3^+^CD4^+^CD25^+^ cells that are TNFR2^+^Foxp3^+^. Naïve mice (*n* = 5–7 per group per time point) received PS50G intratracheally on day 0 or saline as control. Samples were collected on days 1, 3, 7, and 30. **(A)** Stained lung cells were gated on viable CD3^+^CD4^+^CD25^+^ and CD3^+^CD4^+^CD25^−^ cells, followed by gating on TNFR2 co-expressed with Foxp3. Representative FACS contour plots showing TNFR2^+^ cells in the lung on day 3 from PS50G treated mice. Percentages of **(B)** CD3^+^CD4^+^CD25^+^ cells; **(C)** TNFR2^+^Foxp3^+^ regulatory T cells (Treg); **(D)** TNFR2^–^Foxp3^+^ Treg; and **(E)** TNFR2^+^Foxp3^−^ Teff. Data represent the mean ± SEM of at least three experiments. ***p < 0.001.

Intratracheal instillation of PS50G into the lungs of mice increased frequencies of Treg, peaking at day 7, which decreased but remained significantly higher than the saline control group by day 30 (Figures 1A,B). TNFR2^+^ Treg are maximally suppressive (26), and TNFR2^+^ T effector cells (Teff) are maximal cytokine producers (27). PS50G instillation significantly increased the proportion of TNFR2^+^Foxp3^+^ Treg within the total Treg population (CD3^+^CD4^+^CD25^+^) from ~9% at day 1 to ~20% at day 3, peaking at day 7 (>30%), and this increase remained significantly elevated above the saline control group even up to day 30 (Figure 1C). Conversely, TNFR2^−^Foxp3^+^ Treg decreased from day 3 to 7, remaining low to day 30 (Figure 1D). By contrast, PS50G did not change the proportion of TNFR2^+^Foxp3^−^ cells within Teff (CD3^+^CD4^+^CD25^−^ cells) (Figure 1E). The total numbers of TNFR2^+^Foxp3^+^ Treg in the lung also increased following PS50G instillation (Table 1), while the absolute numbers of the other subsets remained unaltered. Overall, PS50G was shown to preferentially promote the induction of TNFR2^+^Foxp3^+^ Treg in the lung.

**Table 1:** PS50G alter the numbers of CD3^+^CD4^+^ T cells, total regulatory T cells (Treg), and tumor necrosis factor 2 cells in the lung and lung-draining lymph nodes (LN).

Cells	Groups	Cell numbers (10^4^) lung	Cell numbers (10^4^)LN
Total cell numbers	Saline	1,054 ± 252	593 ± 207
	PS50G	2,030 ± 162	2,085 ± 109***
CD3^+^CD4^+^ cells	Saline	72.1 ± 5.0	476 ± 29.8
	PS50G	70.2 ± 8.2	867 ± 73.2***
CD3^+^CD4^+^CD25^+^ Treg	Saline	8.84 ± 0.36	59.6 ± 6.1
	PS50G	8.32 ± 2.1	105.8 ± 14.3***
TNFR2^+^Foxp3^+^ within CD3^+^CD4^+^CD25^+^ Treg	Saline	0.87 ± 0.23	9.2 ± 2.3
	PS50G	2.43 ± 0.91***	16.2 ± 2.9***
TNFR2^−^ Foxp3^+^ within CD3^+^CD4^+^CD25^+^ Treg	Saline	5.1 ± 0.9	28.0 ± 6.4
	PS50G	5.01 ± 1.09	54.4 ± 7.21***
CD3^+^CD4^+^CD25^−^ Teff	Saline	60.7 ± 2.87	501.4 ± 20.5
	PS50G	62.3 ± 11.2	902 ± 13.6***
TNFR2^+^Foxp3^−^ within CD3^+^CD4^+^CD25^−^ Teff	Saline	0.89 ± 0.32	11.02 ± 3.3
	PS50G	1.12 ± 0.82	20.09 ± 3.6***

*Naïve mice (*n* = 5–7 per group per time point) received PS50G intratracheally on day 0 or saline as control. Samples were collected on day 3. Lung and lung-draining LN were analyzed for cell numbers and percentages. Data represent the mean ± SEM of at least three experiments*.

***p < 0.001.

### PS50G Instillation Increased the Percentages of TNFR2^+^Foxp3^+^ Treg in the Lung-Draining LN

**Figure 2:**
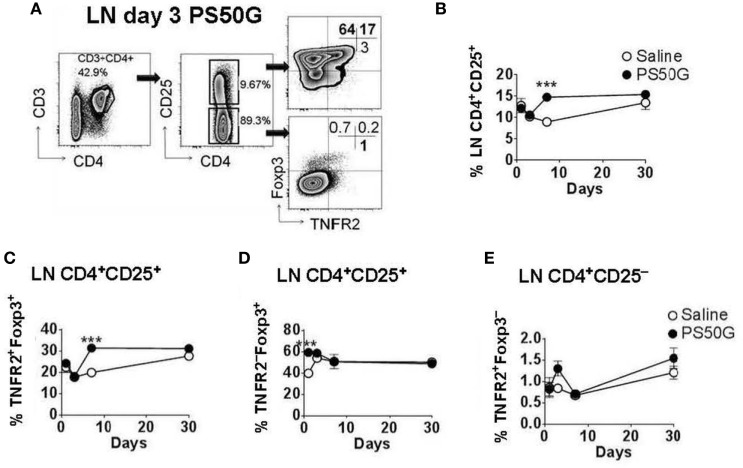
PS50G instillation selectively increases lung-draining lymph nodes (LN) CD3^+^CD4^+^CD25^+^ cells that are TNFR2^+^Foxp3^+^ on day 7. Naïve mice (*n* = 5–7 per group per time point) received PS50G intratracheally on day 0 or saline as control. Samples were collected on days 1, 3, 7, and 30. **(A)** Stained lung-draining LN cells were gated on viable CD3^+^CD4^+^CD25^+^ and CD3^+^CD4^+^CD25^−^ cells, followed by gating on TNFR2 co-expressed with Foxp3. Representative FACS contour plots showing TNFR2^+^ cells in the lung-draining LN at day 3 from PS50G-treated mice. Percentages of **(B)** CD3^+^CD4^+^CD25^+^ cells; **(C)** TNFR2^+^Foxp3^+^ regulatory T cells (Treg); **(D)** TNFR2^–^Foxp3^+^ Treg; and **(E)** TNFR2^+^Foxp3^−^ Teff. Data represent the mean ± SEM of at least three experiments. ***p < 0.001.

While Treg in the lung play a substantial role in controlling lung inflammation, the priming, activation, and expansion of T cells associated with airway inflammation also involves the LN that drain the lungs. To investigate the effects of PS50G on Treg in the lung-draining LN, we applied a similar gating strategy to that used for lung Treg (Figure 2A). Instillation of PS50G did not significantly affect the percentages of CD3^+^CD4^+^CD25^+^ Treg at day 1 and 3, but increased the frequency from ~9 to ~15% at day 7, returning to saline control levels at day 30 (Figure 2B). A similar pattern was observed in the percentages of TNFR2^+^Foxp3^+^ Treg (increasing from ~ 20 to ~ 30% at day 7) (Figure 2C). In contrast, the percentages of TNFR2^–^Foxp3^+^ Treg were significantly increased as early as day 1 and returned to saline control levels from day 3 to day 30 (Figure 2D). PS50G instillation did not affect the frequency of TNFR2^+^Foxp3^−^ Teff, being <2% for all time points in both saline and PS50G groups (Figure 2E). Absolute numbers of CD3^+^CD4^+^ T cells, CD4^+^CD25^+^ Treg, TNFR2^+^Foxp3^+^ Treg, TNFR2^−^Foxp3^+^ Treg, and TNFR2^+^Foxp3^−^ Teff increased on day 3 (Table 1), as reflected by approximately fivefold increase in total lung-draining LN numbers (data not shown). Overall, we saw an increase in the percentages of CD3^+^CD4^+^CD25^+^ Treg, and TNFR2^+^Foxp3^+^ Treg, but not TNFR2^−^Foxp3^+^Treg on day 7 in the lung-draining LN after instillation of PS50G.

### PSG50-Induced Inhibition of AAI Is Associated with Increased Local Efficiency in the Induction of TNFR2^+^Foxp3^+^ Treg Upon Allergen Challenge

**Figure 3:**
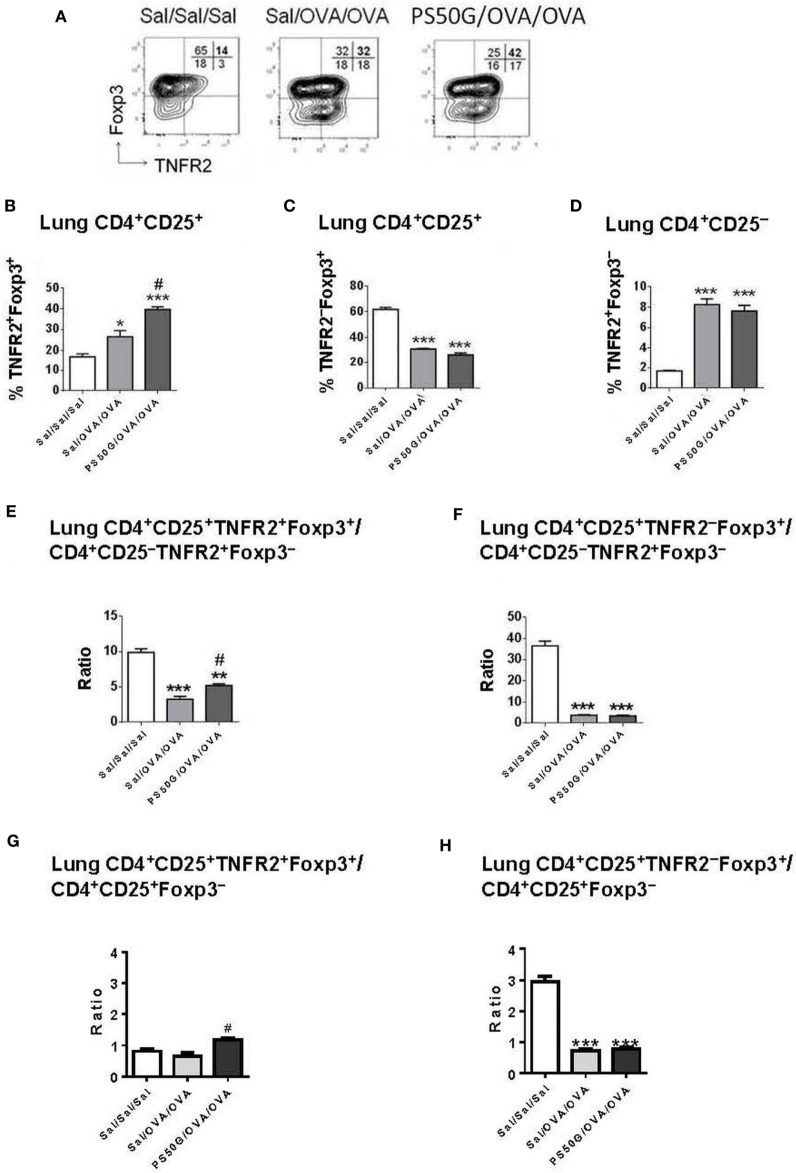
PS50G instillation selectively increases lung CD3^+^CD4^+^CD25^+^ T cells that are TNFR2^+^Foxp3^+^ in allergic airway inflammation mouse model. **(A)** Stained lung cells were gated on viable CD3^+^CD4^+^CD25^+^ and CD3^+^CD4^+^CD25^−^ cells, followed by gating on tumor necrosis factor 2 co-expressed with Foxp3. Percentages of **(B)** TNFR2^+^Foxp3^+^ regulatory T cells (Treg); **(C)** TNFR2^–^Foxp3^+^ Treg and **(D)** TNFR2^+^Foxp3^−^ Teff. Ratios of **(E,G)** TNFR2^+^Foxp3^+^ Treg and **(F,H)** TNFR2^–^Foxp3^+^ Treg to Foxp3^−^ Teff. Data represent the mean ± SEM of at least three experiments with four to six mice per group. *p < 0.05 and ***p < 0.001 compared with saline negative control group (Sal/Sal/Sal); ^#^*p* < 0.01, compared with OVA positive control group (Sal/OVA/OVA).

We previously showed that PS50G instillation inhibits the elicitation of subsequent AAI in atopic mice (8, 9). Herein, we hypothesized that the re-elicitation of Treg, and particularly TNFR2^+^ Treg, by a subsequent allergen challenge, would differ between PS50G vs saline pretreated animals. We also wanted to address whether the proportion of TNFR2^+^ Teff elicited by the allergen would be impacted by prior PS50G exposure. The results showed that PS50G pre-instillation resulted in an increased ability of the lungs to respond by TNFR2^+^Foxp3^+^ Treg upregulation to a subsequent allergen challenge (Figure 3). Specifically, the proportion of TNFR2^+^Foxp3^+^ Treg within total T cells and within Treg was significantly increased in the PS50G/OVA/OVA group compared to the control groups (Sal/Sal/Sal and Sal/OVA/OVA) (Figure 3B). The percentages of lung TNFR2^−^Foxp3^+^ Treg in PS50G/OVA/OVA and Sal/OVA/OVA groups decreased markedly from ~60 to ~30%, an approximately twofold decrease as compared to the saline negative control group (Sal/Sal/Sal) (Figure 3C). On the other hand, the percentages of TNFR2^+^Foxp3^−^ Teff significantly increased after allergen challenge (approximately fourfold), regardless of whether the animals had been pretreated with PS50G or saline (Figure 3D). Thus, PS50G selectively increased TNFR2^+^Foxp3^+^ Treg proportions, without affecting TNFR2^−^Foxp3^+^ Treg or TNFR2^+^Foxp3^−^ Teff, resulting in an increased ratio of TNFR2^+^Foxp3^+^ Treg to Foxp3^−^ Teff compared to the Sal/OVA/OVA group (Figures 3E,G). Furthermore, the ratio of TNFR2^−^Foxp3^+^ Treg to Foxp3^−^ Teff was unchanged compared to the Sal/OVA/OVA group (Figures 3F,H). Given that a portion of the Foxp3^−^ effector cells is CD25^+^, we also analyzed this subset to assure that the ratio of TNFR2^+^Foxp3^+^ Treg to Foxp3^−^ Teff would not be affected. Interestingly, similar patterns were observed for Treg/Teff ratio (Figures 3E–H) indicating that in the lung the total Treg/Teff ratio remains consistent regardless of CD25 expression.

### PSG50-Induced Inhibition of Elicitation of AAI Is Associated with Increased TNFR2^+^Foxp3^+^ Treg in Lung-Draining LN

**Figure 4:**
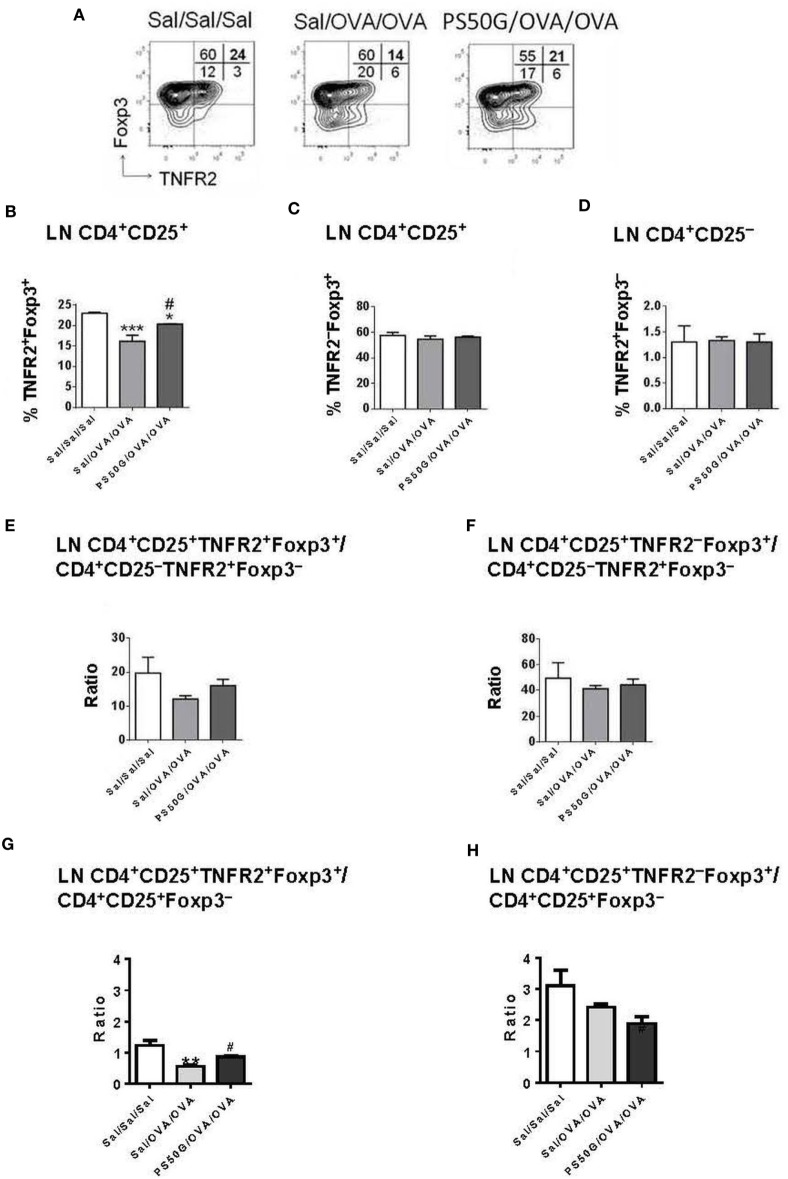
PS50G instillation selectively increases lung-draining lymph nodes CD3^+^CD4^+^CD25^+^ that are TNFR2^+^Foxp3^+^ in allergic airway inflammation mouse model. **(A)** Stained lung cells were gated on viable CD3^+^CD4^+^CD25^+^ and CD3^+^CD4^+^CD25^−^ cells, followed by gating on tumor necrosis factor 2 co-expressed with Foxp3. Percentages of **(B)** TNFR2^+^Foxp3^+^ regulatory T cells (Treg); **(C)** TNFR2^−^Foxp3^+^ Treg; and **(D)** TNFR2^+^Foxp3^−^ Teff. Ratios of **(E,G)** TNFR2^+^Foxp3^+^ Treg and **(F,H)** TNFR2^−^Foxp3^+^ Treg to Foxp3^−^ Teff. Data represent the mean ± SEM of at least three experiments with four to six mice per group. *p < 0.05, **p < 0.01, ***p < 0.001 compared with saline negative control group (Sal/Sal/Sal); ^#^*p* < 0.05, compared with OVA positive control group (Sal/OVA/OVA).

In the lung draining LN, allergen challenge (Sal/OVA/OVA) was followed by a decrease in frequency of TNFR2^+^Foxp3^+^ Treg relative to the saline control group (Sal/Sal/Sal) (Figure 4B). PS50G pretreatment (PS50G/OVA/OVA) prevented this decrease. Although the observed differences were small, the levels of TNFR2^+^Foxp3^+^ Treg were significantly higher in the PS50G/OVA/OVA group (Figure 4B) and showed an increased ratio of CD25^+^TNFR2^+^Foxp3^+^ Treg to CD25^+^Foxp3^−^ Teff (Figure 4G), even though the ratio of CD25^+^TNFR2^+^Foxp3^+^ Treg to CD25^-^TNFR2^+^Foxp3^−^ Teff did not significantly change in any of the groups (Figure 4E). By contrast, no significant differences were observed in the frequencies and ratios of TNFR2^–^Foxp3^+^ Treg to Foxp3^−^ Teff (Figures 4C,F,H). No differences were observed in TNFR2^+^Foxp3^−^ Teff (Figure 4D).

### Mechanisms Underlying the PS50G Induced Increase in TNFR2^+^Foxp3^+^ Treg in the Lung during AAI

**Figure 5:**
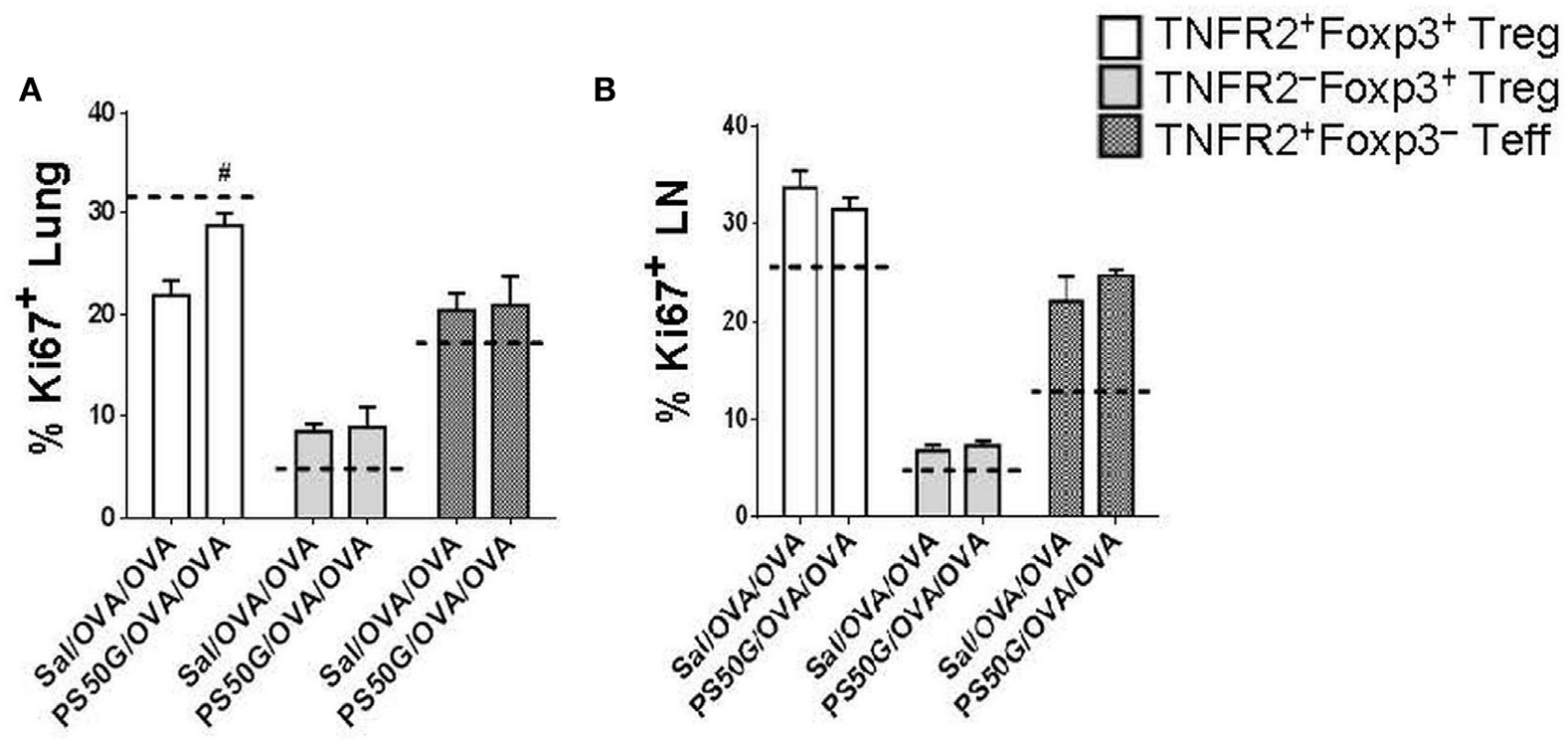
PS50G instillation increases the percentages of Ki67^+^ cells within lung TNFR2^+^Foxp3^+^ regulatory T cells (Treg) during allergic airway inflammation. Stained lung and lung-draining lymph nodes (LN) cells were gated as in Figures 3A and 4A, respectively followed by gating on Ki67. Percentages of **(A)** lung Ki67^+^ and **(B)** lung-draining LN Ki67^+^ within TNFR2^+^Foxp3^+^ Treg; TNFR2^−^Foxp3^+^ Treg and TNFR2^+^Foxp3^−^ Teff. Data represent the mean ± SEM of at least three experiments with four to six mice per group. ^#^*p* < 0.05, compared with OVA positive control group (Sal/OVA/OVA). The dashed lines denote the percentages of cells expressing the respective markers derived from the saline negative control group (Sal/Sal/Sal).

To gain insight into whether the increase in TNFR2^+^Foxp3^+^ Treg proportions and numbers in the lung and lung-draining LN after allergen challenge was driven by increased proliferation, we analyzed expression of the proliferative marker Ki67. PS50G pretreatment (PS50G/OVA/OVA) significantly increased the percentages of Ki67^+^ cells preferentially within TNFR2^+^Foxp3^+^ Treg in the lung post allergen challenge in the AAI model, when compared to Sal/OVA/OVA group (Figure 5A). By contrast, the frequency of proliferated cells within TNFR2^+^Foxp3^−^ Teff in the lung after allergen challenge in mice with a PS50G pretreatment (PS50G/OVA/OVA) were similar to that of the saline negative control group indicated by the dashed line (Sal/Sal/Sal) (Figure 5A). No significant differences in the proportion of Ki67^+^ cells within TNFR2^–^Foxp3^+^ Treg or TNFR2^+^Foxp3^−^ Teff were found between PS50G/OVA/OVA and Sal/OVA/OVA groups, showing that PS50G preferentially induced proliferative expansion of TNFR2^+^Foxp3^+^ Treg. PS50G did not affect the frequency of Ki67^+^ cells for any of the populations examined in the lung-draining LN. However, we observed that the frequency of Ki67^+^ cells within the TNFR2^−^Foxp3^+^ Treg was threefold to fourfold lower than within the TNFR2^+^Foxp3^+^ Treg and TNFR2^+^Foxp3^−^ Teff subsets (Figure 5B). These results suggest that changes in Treg subset frequencies upon PS50G instillation and subsequent allergen challenge increase the TNFR2^+^Foxp3^+^ Treg proliferative state in the lung.

### PS50G Pretreatment Increased LAP^+^ and CTLA-4^+^ Cells within TNFR2^+^Foxp3^+^ Treg in Lung and Lung-Draining LN of AAI Mouse Model

**Figure 6:**
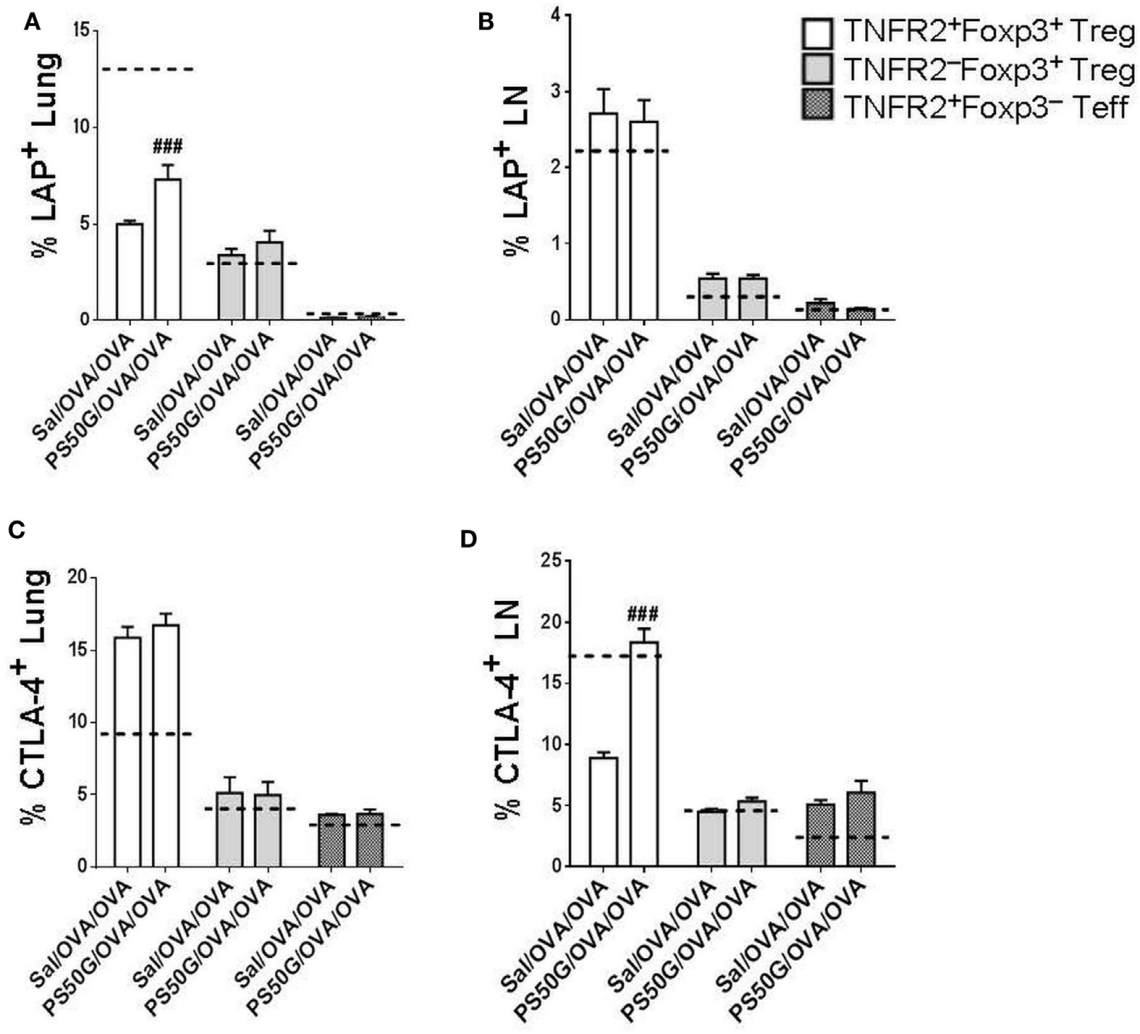
PS50G instillation increases the percentages of lung LAP^+^ and lung-draining lymph nodes (LN) CTLA-4^+^ cells within TNFR2^+^Foxp3^+^ regulatory T cells (Treg) during allergic airway inflammation. Stained lung cells and lung-draining LN cells were gated as in Figures 3A and 4A, respectively, followed by gating on latency associated peptide and CTLA-4. Percentages of **(A)** lung LAP^+^; **(B)** lung-draining LN LAP^+^; **(C)** lung CTLA-4^+^; and **(D)** lung-draining LN CTLA-4^+^ within TNFR2^+^Foxp3^+^ Treg; TNFR2^−^Foxp3^+^ Treg; and TNFR2^+^Foxp3^−^ Teff. Data represent the mean ± SEM of at least three experiments with four to six mice per group. ^###^*p* < 0.001, compared with OVA positive control group (Sal/OVA/OVA). The dashed lines denote the percentages of cells expressing the respective markers derived from the saline negative control group (Sal/Sal/Sal).

Previous studies in other disease models have shown that Treg have maximal suppressive capacity, which is associated with higher expression of immunosuppressive molecules such as CTLA-4 (28, 29). To confirm that the TNFR2^+^Foxp3^+^ Treg elicited during AAI could also exhibit a suppressive potential, we analyzed their expression of the TGF-β binding molecule LAP and of CTLA-4. Moreover, we tested whether PS50G pre-exposure could further alter the expression of these functional molecules on the TNFR2^+^Foxp3^+^ Treg. We found that PS50G treatment significantly increased the proportion of LAP^+^ cells within the TNFR2^+^Foxp3^+^ Treg subset, but not within the TNFR2^−^Foxp3^+^ Treg and TNFR2^+^Foxp3^−^ Teff subsets in the lung (Figure 6A). PG50G did not alter the proportion of LAP^+^ cells in any cell population in the lung-draining LN (Figure 6B). Consistent with the finding that LAP expression is not associated with Teff, we identified the lowest frequency of LAP^+^ cells within TNFR2^+^Foxp3^−^ Teff both in the lung and in the lung-draining LN (<0.3%) (Figures 6A,B). Although PS50G did not alter the proportion of CTLA-4 positive Treg (TNFR2^+^Foxp3^+^ Treg and TNFR2^−^Foxp3^+^ Treg) in the lung (Figure 6C), they induced a twofold increase in the frequency of CTLA-4^+^ cells exclusively within TNFR2^+^Foxp3^+^ Treg in the lung-draining LN (Figure 6D). Therefore, PS50G increased expression of molecules associated with the suppressive function of TNFR2^+^Foxp3^+^ Treg in the lung and lung-draining LN.

### Activation of CD103^+^ DC Positively Correlates with TNFR2^+^Foxp3^+^ Treg Expansion

**Figure 7:**
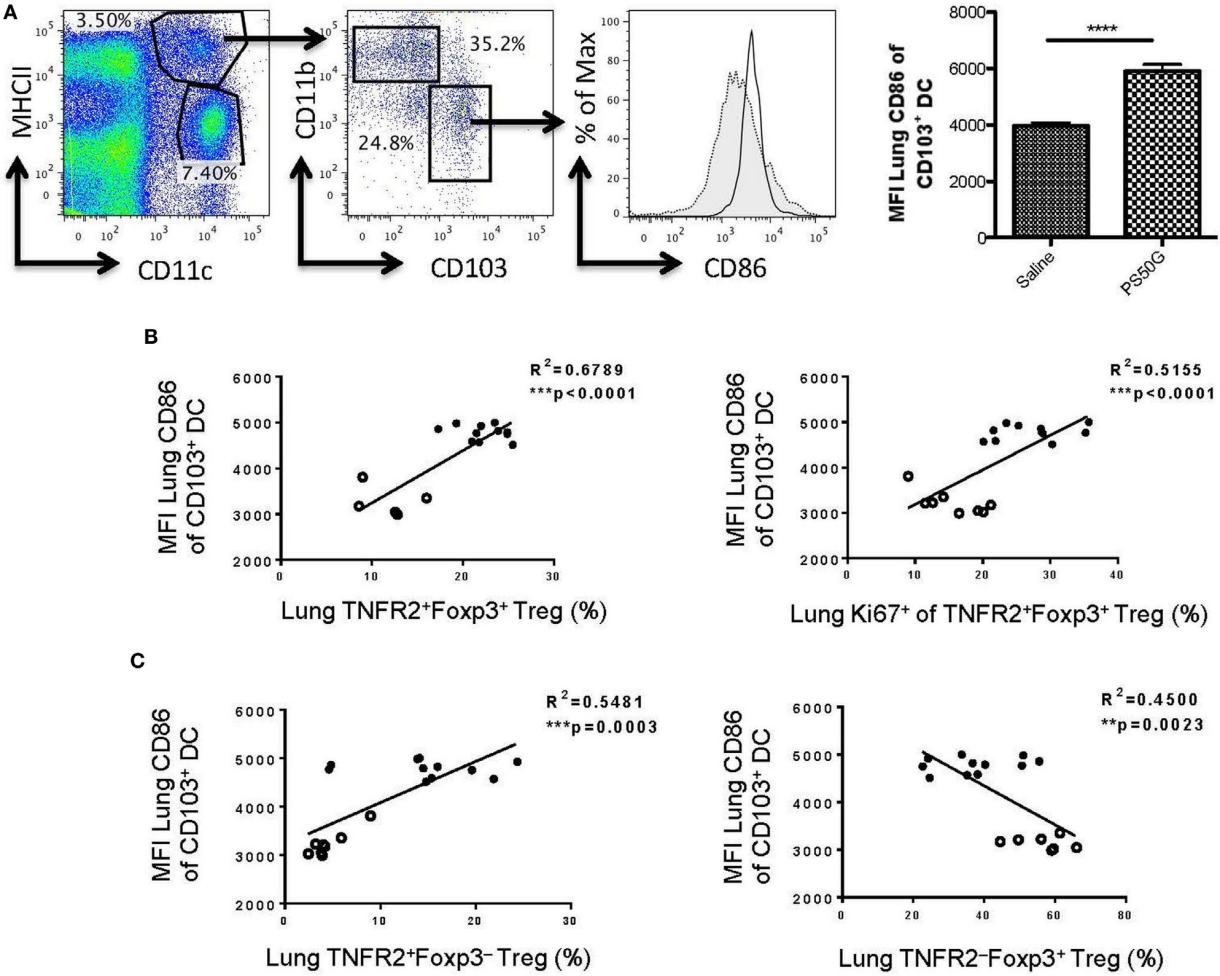
CD86 expression on lung CD103^+^ dendritic cell (DC) positively correlates with the proportions of TNFR2^+^ regulatory T cells (Treg) and TNFR2^+^Foxp3^+^ that are expanded (Ki67^+^). Naïve mice (*n* = 5–7 per group per time point) received PS50G i.t on day 0 or saline as control. Samples were collected on days 1, 3, 7, and 30. **(A)** Stained lung cells were gated on viable MHCII^high^CD11c^+^, followed by gating on CD11b^−^CD103^+^ cells on day 3 post instillations. Expression of CD86 population in saline (gray line, filled histogram) and PS50G (black line, open histogram). MFI of MHCII^high^CD11c^+^CD11b^−^CD103^+^ DC on day 3 post instillations. Data represent the mean ± SEM. ***p < 0.001 of at least three experiments. The percentages of **(B)** TNFR2^+^Foxp3^+^ Treg (left panel), TNFR2^+^Foxp3^+^Ki67^+^ Treg (right panel), and **(C)** TNFR2^+^FoxP3^−^ Treg (left panel) positively correlated with MFI of CD86 on CD103^+^ DC (MHCII^+^CD11c^+^CD11b^−^). The percentages of **(C)** TNFR2^−^FoxP3^+^ Treg (right panel) negatively correlated with CD103^+^ DC. Open circles correspond to saline group (*n* = 5–8) and closed circles correspond to PS50G (*n* = 11) data partially from days 1, 3, 7, and 30.

Our previous data showed that the expression of CD86 on CD103^+^ DC is positively correlated with PS50G uptake (9). To investigate the possible relationship between PS50G uptake by tolerogenic CD103^+^ DC and Treg in the lung, we analyzed the correlation between activated CD103^+^ DC (based on CD86 expression) with TNFR2^+^Foxp3^+^ Treg. As predicted, PS50G increased the frequency of activated CD103^+^ DC (Figure 7A), which positively correlated with overall increases of TNFR2^+^ Treg (Figures 7B,C, left panel) and the proportion of TNFR2^+^Foxp3^+^ Treg that proliferated after PS50G administration (based on Ki67 expression) (Figure 7B, right panel). By contrast, activated CD103^+^ DC negatively correlated with TNFR2^−^Foxp3^+^ Treg (Figure 7C, right panel). Together this data suggest that CD103^+^ DC activation promotes the expansion of highly proliferative TNFR2 expressing Treg.

## Discussion

A number of ambient, anthropogenic, and ENP have been described that exert detrimental effects on lung immune homeostasis and exacerbate the symptoms of asthma and lung inflammation upon allergen challenge in susceptible individuals (30, 31). However, our studies suggest a radically different role for non-toxic particles such as PS50G: promoting homeostasis and preventing the elicitation of inflammation upon allergen challenge in atopic individuals (8, 9). Our initial studies suggested that PS50G can modulate pulmonary DC function (8, 9). Here, we reveal a novel role for PS50G, leading to augmented elicitation of lung TNFR2^+^Foxp3^+^ Treg upon allergen challenge in sensitized animals, associated with increased control of allergic lung airway inflammation. Furthermore, for the first time, we show that NP can be engineered to induce the upregulation of TNFR2^+^Foxp3^+^ Treg in the lung.

Increased frequencies of Treg in the periphery or lymphoid organs indicate that Treg have either proliferated (32, 33) or migrated into the tissue (34, 35). Previously, it was shown that the size of the Treg pool is critical for maintaining immunological balance, and even relatively minor modulation of Treg numbers alters immunity, with preferential effects on T helper 2 (Th2) immunity (36). Our present data are in agreement with Tian et al., showing efficient prevention of Th2 cell elicitation by allergens in the lung after allergen challenge. Chen et al. have demonstrated that, in peripheral lymphoid organs, Treg and Teff expressing TNFR2 exhibit greater proliferative capacity than the non-TNFR2 expressing subsets (27). Here, we show for the first time that, in the lung, even in a largely non-inflammatory environment (during homeostasis), TNFR2^+^Foxp3^+^ Treg and TNFR2^+^Foxp3^−^ Teff both exhibit a greater proliferative capacity. Therefore, TNFR2^+^Foxp3^+^ Treg with strong proliferative capacity in the lung may be critical to respond rapidly to inflammatory stimuli in this environment, in addition to effectively controlling the activation of Th2 effectors. Previous studies have shown that only TNFR2^+^Foxp3^+^ Treg are able to suppress highly bioactive TNFR2^+^Foxp3^−^ Teff (26), which we observed are also highly elicited upon allergen challenge. Therefore, TNFR2^+^Foxp3^+^ Treg are a pivotal determinant of the level and type of immunity elicited in response to inflammatory environmental stimuli in the lung. Overall, our findings suggest that TNFR2^+^Foxp3^+^ Treg proliferate in the lung to maintain homeostasis and limit inflammatory responses, while maintaining “appropriate” responses to harmless airborne stimuli.

After allergen challenge in atopic animals, the proliferation of TNFR2^+^Foxp3^+^ Treg in the lung was increased in animals that had been previously pretreated with PS50G. CTLA-4 expression in LN, but not the periphery, is critical to prevent elicitation of adaptive immunity (37). In turn, LAP expression is associated with potent peripherally activated Treg immunosuppressive phenotypes (38). Pretreatment with PS50G resulted in higher levels of expression of LAP (in the lung) and CTLA-4 (in the lung-draining LN) specifically on TNFR2^+^Foxp3^+^ Treg, suggesting they can promote increases in the relevant, organ-specific, maximally suppressive phenotypes. Thus, the increases in TNFR2^+^Foxp3^+^ Treg during AAI, promoted by PS50G pretreatment, were associated with their increased proliferative expansion capacity. Together, these results show how PS50G can increase the long-term capacity of the lungs to maintain a normal response following an allergen challenge. In addition, the response occurred without allergic Th2 driven exacerbations even in atopic animals, by preferentially expanding TNFR2^+^Foxp3^+^ Treg. A recent study further demonstrated the critical nature of TNFR2 driven regulation in the lung, by showing that aberrant TNFR2 signaling exacerbates airway inflammation in an AAI mouse model, specifically by promoting Th2 and Th17 cell polarization while inhibiting Th1 and CD4^+^CD25^+^ T cells differentiation (39). In this context, PS50G may improve the capacity of the lungs to control inflammation through TNFR2 signaling. In addition, as shown by our results, PS50G can also increase the TNFR2^+^Foxp3^+^ Treg pool and its proliferative potential. The latter may support the maintenance of an effective immunoregulatory pool size for homeostatic control in inflammatory environments.

Previously, we demonstrated that PS50G are preferentially taken up by DC in the lung and may affect their long-term function. The present study extends these findings by showing that PS50G increase the frequency and enhance the suppressor phenotype of TNFR2^+^Foxp3^+^ Treg. Such a broad immunological imprint has important consequences on adaptive immune responses in the lung, especially in controlling allergen-induced Th2 cells in AAI. How different subsets of effector T cells, relevant to diverse lung diseases, ranging from Th2 cells in inflammatory allergic diseases, to Th1 and Th17 cells in cancers, are affected by TNFR2^+^Foxp3^+^ Treg expansion in the lung induced by particles such as PS50G or other stimuli, will be a useful question to address in future studies in diverse lung disease models. From a practical point of view, such properties need to be understood if NP is to be rationally deployed as carriers to deliver drugs and vaccines into the lungs (2). Indeed, like PS50G particles, gold NP have more recently been observed to promote homeostatic imprints in the lung (40) and silver NP for the overall homeostasis of the intestinal tract (41). Conversely, toxic and pro-inflammatory NP such as those derived from diesel exhaust fumes promote increased susceptibility to allergic airways inflammation (42). Although the immunological basis of such imprints was not explored in many of these studies, we speculate that DC functional impairment and altered TNFR2^+^Foxp3^+^ Treg function are likely to be critically involved.

Tolerogenic and migratory CD103^+^ DC travel toward lung-draining LN to prime the differentiation of naïve CD4^+^ T cells into Treg (17), whereas lung macrophages are involved in maintaining Foxp3 expression by Treg, once these cells populate the lung tissue (21). Our data support previous findings on CD103^+^ DC in establishing airway tolerance (17–19) and further suggest that CD103^+^ DC might promote TNFR2^+^Foxp3^+^ Treg expansion. As this is the first study investigating the effects of non-toxic ENP on TNFR2^+^Foxp3^+^ Treg in the lung, further studies should follow to evaluate the role of CD103^+^ DC in priming and/or inducing TNFR2^+^Foxp3^+^ Treg both in the lung and lymphoid organs.

While PS50G were used as a new model to show that TNFR2^+^Foxp3^+^ Treg can be preferentially expanded in the lung, these NP are not biodegradable, which may complicate their direct clinical translatability. Nevertheless, our previous studies have shown that PS50G are biocompatible, non-toxic and non-inflammatory even at high doses in the lung. While conventional materials such as nickel or titanium oxide cannot be used at high doses in the lung given toxicity concerns (43, 44), the development of new types of biodegradable NP may open to door to new classes of immunoregulators to help control inflammatory lung disease. In this context, some polymers such as poly(lactide-*co*-glycolide) (3) and fullerenes (45) hold significant promise and biodegradable NP of a larger (46) or smaller size (47, 48) have already been shown to suppress inflammation and to induce Treg. Furthermore, distinct nanoparticle sizes may induce different anti-inflammatory responses according to the type of disease. For example, Miller and colleagues showed that intravenous infusion of poly(lactide-*co*-glycolide) microparticles (500 nm) promote anti-inflammatory effects in the periphery in mice with relapsing experimental autoimmune encephalomyelitis (46). However, our previous studies in the lung showed that PS50G (50 nm) particles are capable of providing a broader anti-inflammatory imprint than PS500G (500 nm) particles, potentially associated by their preferential uptake by DC (rather than macrophages), including CD103^+^ DC. In future studies, it will be important to map the extent to which particle size preferences will influence anti-inflammatory activity in diverse peripheral inflammation models.

Collectively, the present study proposes a novel mechanism by which NP such as PS50G, modulate the adaptive immune response by altering TNFR2^+^Foxp3^+^ Treg homeostasis, thereby decreasing susceptibility to allergic disease. In summary, together with our previous findings, these results implicate an important role of non-toxic ENP in establishing and preserving Treg numbers and functions through mechanisms such as (a) increasing the proliferative rate of Treg, thus altering the ratio between Treg and Teff, (b) maintenance of a self amplification loop by TNF/TNFR2 interaction, and (c) indirectly targeting CD103^+^ DC to modify Treg.

## Ethics Statement

This study was carried out in accordance with the recommendations of Victorian Prevention of Cruelty to Animals Act 1986 and its incorporated Australian code of practice for the care and use of animals for scientific purposes 2013. The protocol was approved by the Alfred Medical Research and Education Precinct (AMREP) animal ethics committee.

## Author Contributions

RM, JL, JLS and CH performed experiments and analyzed data; MP, CH, RO, and JR supervised RM and JL. MP, CH designed experiments and analyzed data; MP, CH, RO, and JR designed the overall project; RM, MP, CH, and JB interpreted data and wrote the manuscript; MP, CH, JB, JR, and RO edited the manuscript. MP, CH, JR, and RO provided funding for the project. CH and MP contributed equally.

## Conflict of Interest Statement

The authors declare that the research was conducted in the absence of any commercial or financial relationships that could be construed as a potential conflict of interest.
